# Genome-Wide Identification and Comparative Analysis of *RALF* Gene Family in Legume and Non-Legume Species

**DOI:** 10.3390/ijms24108842

**Published:** 2023-05-16

**Authors:** Yancui Jia, Youguo Li

**Affiliations:** State Key Laboratory of Agricultural Microbiology, College of Life Science and Technology, Huazhong Agricultural University, No. 1 Shizishan Road, Hongshan District, Wuhan 430070, China; yancuijia@126.com

**Keywords:** legume, RALF, phylogenetic analysis, motifs/residues, expansion, nodule symbiosis

## Abstract

Rapid alkalinization factor (RALF) are small secreted peptide hormones that can induce rapid alkalinization in a medium. They act as signaling molecules in plants, playing a critical role in plant development and growth, especially in plant immunity. Although the function of RALF peptides has been comprehensively analyzed, the evolutionary mechanism of RALFs in symbiosis has not been studied. In this study, 41, 24, 17 and 12 *RALFs* were identified in *Arabidopsis*, soybean, *Lotus* and *Medicago*, respectively. A comparative analysis including the molecular characteristics and conserved motifs suggested that the RALF pre-peptides in soybean represented a higher value of isoelectric point and more conservative motifs/residues composition than other species. All 94 RALFs were divided into two clades according to the phylogenetic analysis. Chromosome distribution and synteny analysis suggested that the expansion of the *RALF* gene family in *Arabidopsis* mainly depended on tandem duplication, while segment duplication played a dominant role in legume species. The expression levels of most *RALFs* in soybean were significantly affected by the treatment of rhizobia. Seven *GmRALFs* are potentially involved in the release of rhizobia in the cortex cells. Overall, our research provides novel insights into the understanding of the role of the *RALF* gene family in nodule symbiosis.

## 1. Introduction

Dynamically regulating extracellular pH is necessary for maintaining normal physiological activities in plants [1]. Extracellular alkalization in plants refers to the increase in pH of the apoplast, which is a common response to the stress and pathogen invasion [2,3,4,5,6]. Previous studies have indicated that extracellular alkalinization is the earliest recordable response in pattern-triggered immunity (PTI) [2,3,5,6]. It induces the change in reactive oxygen species (ROS), nitric oxide, Ca^+^ oscillations and ion movement and the inhibition of H^+^-ATPase [2,3,5,6]. Nodulation factors (NFs) also triggered extracellular alkalization, which is a hallmark of the early symbiotic relationship established between rhizobia and legume plants [7,8]. Additionally, extracellular alkalization can affect the physical properties of cell wall, the interaction between plants and pathogens and the activity of pathogenicity factors secreted by the pathogen. For example, extracellular alkalization could promote the accumulation and release of antimicrobial peptides (AMPs) in the cell wall, thereby enhancing the plant’s antibacterial activity against pathogens [9,10,11]. Alkaline stress inhibits the infection rate of plants and the growth rate of pathogens [12]. Importantly, the apolastic pH is one of the major ambient traits affecting the activity of pathogenicity factors secreted by the pathogen, such as one pectate lyase (PL) produced by *Colletotrichum gloeosporioides*, which is a key virulence factor in disease development [4,13].

Small peptides are important extracellular signals for cell-to-cell communication in plants, as they participate in plant growth, development, and the interaction between the host and pathogen [14,15]. The symbiotic nitrogen (N)-fixation system formed by legume and rhizobia fixed up to 200 million tonnes of N per annum, which plays a crucial role in N fixation and cycling [16]. According to the previous study, small peptides are known to be important in this process, such as C-terminally encoded peptides (CEP), early nodulin 40 (ENOD40), phytosulfokine (PSK), nodule cysteine rich (NCR), glycine-rich peptides (GRP), small nodulin acidic RNA-binding peptides (SNARP), which have a positive effect on nodulation, as well as rapid alkanization factor (RALF), DLV, CLV3/ESR related (CLE), which have a negative effect on nodulation [17,18,19,20,21,22,23,24]. Among these small peptides, a secreted small peptide, RALF, is involved in multiple polar growth processes, such as pollen tube and root hair growth [25,26,27,28,29,30]. However, it is not clear whether RALF is involved in the invasion of rhizobia (the polarized growth of infection thread during symbiotic).

RALF peptides belong to the cysteine-rich peptide family which can induce rapid alkalinization in cell suspension cultures [31]. Previous research has shown that *RALFs* are widely distributed in plants, animals and fungi [30,32,33,34,35], and played an important role in regulating diverse physiological processes, such as the pollen tube development, root and root hair growth, immune response and nodulation [24,26,27,28,29,31,35,36,37,38,39,40,41]. For example, AtRALF4/9 interacted with ANXURA (ANX1), ANX2 and BUDDHA’S PAPER SEAL (BUPS) 1 and BUPS2 complex to maintain integrity during the growth of pollen tube, but at the interface of pollen tube–female gametophyte contact, AtRALF4/9 was replaced by AtRALF34 caused a deregulating BUPS-ANXUP signaling and release the sperm [27]. AtRALF34 can also interact with THESEUS1 (THE1) and play a role in the fine-tuning of lateral root initiation [29]. In addition, AtRALF1, AtRALF22 and AtRALF23 can interact with *Catharanthus roseus* RLK1-LIKE (CrRLK1L) receptor kinases, FERONIA (FER), to regulate root growth, abiotic and biotic stress responses, respectively [38,42,43]. RALFs encoded by fungi induce a rapid alkalinization, which increases the infection ability in their host [32]. In the communication between soybean and nematodes, either independent horizontal transfers or the convergent evolution of RALFs has been found. For example, the nematode-encoded RALF peptide, MiRALF1, can bind to plant FERONIA to increase the parasitism [44]. However, a malectin-like receptor kinase in soybean, GmLMM1, specifically binds to MiRALF1 and suppresses plant immunity [35]. The effects on nodulation of *RALFs* are diverse during the symbiotic nitrogen fixation process. For example, RALF had a negative effect on the number of infection threads and nodules in *Medicago* but played a positive effect on the nodule number in 0 mM nitrate [24,45].

The primary structure of RALF precursor contains an N-terminal signal peptide and several conserved motifs or residues, including a conserved dibasic site (RR motif) upstream of the active peptide which is essential for proper maturation and release of RALF active peptides; a -YIXY- motif which is important for binding to the receptor and alkalinization activity; a leu-11 (Leucine at position 11) at the N terminus of the mature peptide sequence which is important for alkalinization activity; and four cysteines at the C-terminal which has been shown to be involved in forming disulfide bridges and are important for protein conformation [31,46,47,48,49]. Although the sequence conservation of the N-terminal signal peptide is lower in plants, the precursor that contains a signal peptide may be a secreted protein [46,50]. The RR motif can be recognized and cleaved by site-1 protease (S1P), resulting in the production of an active peptide in *Arabidopsis* [50]. When the first Arg is mutated to Ala in the AtRAL1 precursor, no processed peptide is detected in plants [47]. Therefore, the production of active peptides is dependent on the existence of RR motif. Another conserved motif is -YIXY- motif, which is essential for RALF1-induced alkalinization [48] and RALF23-induced LLG1-FER heterodimerization, immunity and growth inhibition [39]. The second amino acid after -YIXY- motif is Leu-11, which is an important component of the carboxyterminal end of RALF that probably maintains the proper spatial arrangement [48]. The four conserved cysteine residues at C-terminal can form disulfide bridges and play a crucial role in the correct folding of the mature RALF protein [49].

Genome-wide identification and phylogenetic analyses of *RALFs* family in different species can help us to reveal the evolutionary history of RALF, determine its function and provide new resources for further research. Up to now, a total of 40 and 13 *RALFs* have been identified in the model plants *Arabidopsis* and *Medicago*, respectively [51,52,53]. 43, 34 and 23 *RALFs* have been identified in the important economic and crop plants, such as rice, corn and soybean, respectively [51,52,53,54]. The evolutionary analysis of the *RALF* gene family revealed that tandem duplication played a dominant role in the expansion [54]. Although studies on RALF have revealed the role of the *RALF* gene family in regulating plant growth and development [55,56], but the regulatory mechanisms of the *RALF* gene family in symbiosis between legume and rhizobia are still poorly reported [24,45].

To gain insight into the role of the *RALF* gene family in legume species and explore their role in symbiosis, a comparative analysis of *RALF* gene family between non-legume plant (*Arabidopsis*) and legume plants (soybean, *Lotus* and *Medicago*) was performed, including the molecular characteristics, conserved motifs, phylogenetic classifications, chromosome distribution and synteny analysis. Furthermore, the expression patterns of *RALFs* were systematically investigated in flowers, root hairs and nodules after non-inoculated and inoculated treatment. This study is the first report on the exploration of the *RALF* gene family in symbiosis which will facilitate the functional study of *RALF* gene family in symbiosis.

## 2. Results

### 2.1. Identification and Molecular Characterization of RALFs in Legume and Non-Legume Genomes

To extensively identify legume *RALFs*, a total of 41 AtRALF precursor protein sequences, including thirty-seven RALFs confirmed in a previous study [57] and four new AtRALF protein sequences identified in our study, were used as seed sequences to search against the genome of soybean [58], *Lotus* [59] and *Medicago* [60]. After eliminating incomplete and redundant sequences, the remaining sequences were submitted to The Conserved Domain Database (CDD), Pfam and Simple Modular Architecture Research Tool (SMART) database to ensure that they contained the RALF domain. A total of 53 legume *RALFs* were identified, including 24 *GmRALFs*, 17 *LjRALFs* and 12 *MtRALFs*. It is worth mentioning that the *RALF* gene number in legume genomes was far less than that of *Arabidopsis*, although the legume plants had a lager genome sizes, chromosome numbers and total gene numbers than *Arabidopsis* (Table S1). This indicated that the *RALF* genes experienced relatively large differentiation in the process of evolution resulting in a clear division and single function of *RALF* gene family [61]. Then, these genes were named as *GmRALF1* to *GmRALF24*, *LjRALF1* to *LjRALF17* and *MtRALF1* to *MtRALF13*, respectively, according to their order on the corresponding chromosomes, and the four new *RALFs* in *Arabidopsis* were named follow the other members (Table S2).

Basic molecular information of all the 94 *RALFs*, including the gene name, length of DNA, cDNA, protein, molecular weight (MW), isoelectric point (pI) value and subcellular location were presented in Table S3. A physicochemical property analysis showed that the amino acid and molecular weight of RALF precursor protein varied largely from 64 aa/7.21 kDa (AtRALF37) to 321 aa/34.96 kDa (AtRALF38) in *Arabidopsis*, 53 aa/6.04 kDa (GmRALF8) to 174 aa/19.73 kDa (GmRALF3) in soybean, 66 aa/7.46 kDa (LjRALF8) to 138 aa/15.58 kDa (LjRALF17) in *Lotus* and 69 aa/7.54 kDa (MtRALF4) to 135 aa/15.44 kDa (MtRALF1) in *Medicago*, respectively. The average pI values of RALF precursor protein in the four species were 8.98 (*Arabidopsis*), 8.84 (soybean), 8.79 (*Lotus*) and 8.41 (*Medicago*). The minimum and maximum pI were 5.01 and 10.31 in *Arabidopsis*, and all soybean RALF precursor proteins have a pI above 7.00, even though they had a smaller pI variation range from 7.70 to 10.09. The predicted subcellular localization of *RALFs* showed that most of the *RALFs* were localized in extracellular regions, and only a few genes were localized in pollen tube, chloroplast, mitochondria and nucleus.

### 2.2. Phylogenetic Relationships and Classification of 94 RALF Family Genes

**Figure 1:**
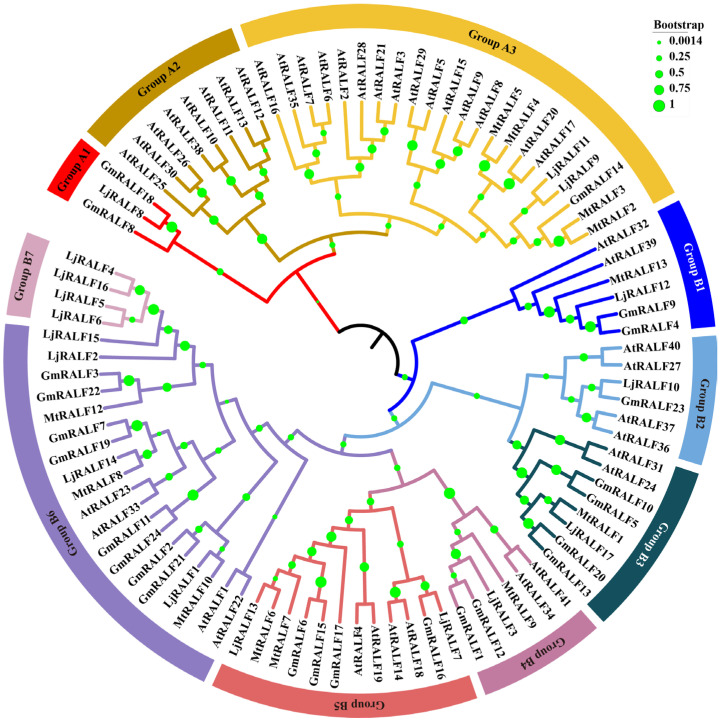
Phylogenetic relationships of 94 RALFs from non-legume and legume species. The unrooted tree was constructed with the MEGA 6.0 software using 94 full-length RALF protein sequences (41, *At*; 24, *Gm*; 12, *Lj*; 17, *Mt*) by the neighbor-joining method with 1000 bootstrap replicates. Bootstrap values are shown in different-sized solid circles on the branches. Different line colors represent different subgroups. Species acronym used: *At*, *Arabidopsis thaliana*; *Gm*, *Glycine max*; *Lj*, *Lotus japonicus;* and *Mt*, *Medicago truncatula*.

In order to better reveal the evolutionary relationships of the RALF proteins from legume and non-legume species, an unrooted tree was constructed by using a full-length RALF protein (Figure 1). According to the tree topology and support values, a total of 94 *RALFs* were separated into two main groups: Group A and Group B. These two groups all contained *Arabidopsis*, soybean, *Medicago* and *Lotus RALFs*, suggesting that the RALF family proteins have evolutional conservation. According to the statistical results, Group A was mainly composed of *Arabidopsis RALFs*, while Group B contained most of the legume *RALFs* (Table S4). Group A was further divided into three subgroups (Group A1–A3) and Group B was further divided into seven subgroups (Group B1–B7), respectively. Most subgroups contained legume and non-legume *RALFs* except for three groups: Group A1, Group A2 and Group B7. Group A1 was the smallest subgroup with two *GmRALFs* and one *LjRALFs*, and Group B7 only contained four *LjRALFs*. By contrast, Group A2 contained eight *AtRALF* genes merely from non-legume species. Evolutionary studies indicated that Brassicales appeared later than Fabales [61]. Therefore, the genes in Group A2 may be new genes generated in evolution.

In order to further reveal the functional characteristics of each group, a statistical analysis was made of the conserved motifs/residues, including signal peptide at the N-terminal, RR motif, YI/LXY motif, Leu-11 and four conserved cysteine at the C-terminal (Table S5). Group A showed poor conservation of the main functional motifs/residues, such as the absence of the RR motif, and less conservation of YI/LXY motif and Leu-11 residue in all groups. However, in Group B, five out of seven subgroups (Group B1, Group B3, Group B4, Group B5, Group B6) showed higher conservation of all the main functional motifs/residues, apart from Group B2 and Group B7.

Despite all the above, it was worth noting that in the evolutionary tree, the *RALFs*, especially from single species, had a tendency to join together and showed a closer relationship. The obvious clustering phenomenon maybe indicate that *RALFs* have functional differentiation among different species.

### 2.3. Conserved Motifs and Residues Analysis of 94 RALFs

The RALF proteins have many functional motifs/residues such as the signal peptide at N-terminal, dibasic site (RR motif), YI/LXY motif, Leu residue at position 11 (Leu-11) and four conserved cysteines at C-terminal according to a previous study [54]. To gain insight into the structural features of RALF proteins in different species, all 94 RALF protein sequences were used (Table S6). In this study, signal peptides were predicted in 39 out of 41 AtRALFs, 19 out of 24 GmRALFs and all RALFs of *Lotus* and *Medicago*. In addition, GmRALF1 and GmRALF3 contained chloroplast transit peptide and mitochondrial targeting peptide in the N-terminal, respectively, not common signal peptide sequence.

The RR motif can be recognized and digested by the protease to produce a mature RALF peptide to acquire rapid alkalinization of cell culture media [31,47]. The statistical results indicated that the RR motif had the highest proportion in soybean (83.33%, 20/24), followed by in the *Medicago* (66.67%, 8/12) and *Lotus* (52.94%, 9/17) (Table 1). Surprisingly, the proportion of RR motif in *Arabidopsis*, a non-legume species is only 29.27% (12/41), which means that most of the RALF precursor proteins cannot be processed to produce mature and active peptides in *Arabidopsis*. 

Considering that a conserved dibasic site (RR) is essential for processing active and functional RALF peptide [47,50,52]; then, a statistic analysis of the other motifs/residues on maturate RALF peptide was performed under the presence of the RR motif. As shown in Table 1, the proportion of YI/LXY and Leu-11 in soybean was also the highest, reaching 70.83% (17/24) and 83.33% (20/24), followed by 58.33% (7/12) and 66.67% (8/12) in *Medicago*, 47.06% (8/17) and 52.94% (9/17) in *Lotus*. Expectedly, *Arabidopsis* showed a poor conservation on these two main alkalinizing activity regions (29.27% (12/41) and 26.83% (11/41)). Similar results appeared in the statistical results of four conserved cysteines. According to a previous study, cysteine residues at C-terminal can form disulfide bridges and played a crucial role in the correct folding of mature RALF protein [49]. The higher conservation of four cysteine residues in legume plants, especially in soybean, implied that legume RALFs were more likely to form three-dimensional (3D) structures and to perform normal functions [39].

In summary, the functional motifs/residues of the RALF precursor proteins showed higher conservation in legume plants, especially in soybean, compared with *Arabidopsis*. The significant differences in motifs/residues conservation among different species imply the functional strength of the RALFs. However, whether this indicates that RALFs in legume plants participate in biological processes different from those in non-legume plant remains to be studied.

### 2.4. Chromosome Distribution and Synteny Analysis of RALF Family Genes

**Figure 2:**
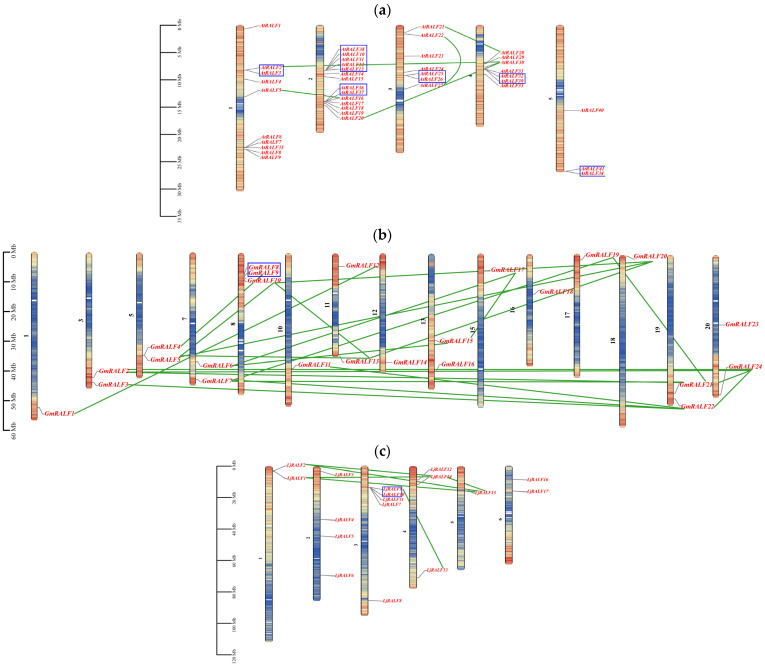

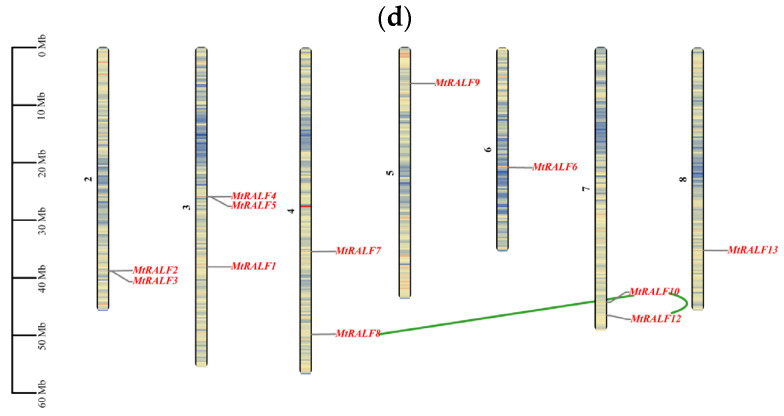
Chromosome distribution of non-legume and legume *RALFs*. (**a**) The distribution of 41 *AtRALFs* on *Arabidopsis* chromosomes. (**b**) The distribution of 24 *GmRALFs* on soybean chromosomes. (**c**) The distribution of 17 *LjRALFs* on *lotus* chromosomes. (**d**) The distribution of 12 *MtRALFs* on *Medicogo* chromosomes. The scale on left was in megabase (Mb). The names of chromosome were placed at the left. Gene names were to the right of the chromosomes. The blue box showed the tandem duplication genes pairs. The green lines represented segment duplication gene pairs. Gradient colors from red to blue that attached to chromosomes were corresponding from high to low gene density by setting a suitable hereditary interval. The hereditary interval of *Arabidopsis*, soybean, *lotus* and *Medicago* were 100 kb, 500 kb, 500 kb and 300 kb.

To investigate the relationships between genetic divergence and gene duplications of *RALFs*, a chromosome distribution was constructed with the help of the TBtools software (Figure 2). The results showed that all *RALF* family members of *Arabidopsis* and *Lotus* were distributed unevenly on all the chromosomes, while *RALF* family members of soybean or *Medicago* had a distribution on 15 of 20 and 7 of 8 chromosomes, respectively. In addition, most of the *RALFs* were located in regions with high gene density among four species. 

The appearance of tandem duplication and segment duplication in the genome was regarded as a major driving force in plant gene family expansion [62]. In order to understand the mode of *RALF* gene family expansion, this study performed an analysis on the duplication events of *RALF* genes within the species. The results suggested that on the genome of *Arabidopsis*, soybean, *Lotus* and *Medicago*, there were 6, 1, 1, 0 tandem duplications and 5, 22, 6, 2 segment duplications, respectively. Most segment duplication events occurred in legume genomes, and largest tandem duplication occurred in *Arabidopsis* genomes, indicating that the expansion of the *RALF* gene family in *Arabidopsis* mainly depends on tandem duplication, while the expansion of the *RALF* gene family in legumes mainly depends on segment duplication, this is consistent with previous studies [63]. These results suggested that the expansion of *RALF* members in different species through distinct mechanisms after they separated from their ancestors. 

Further analysis of the protein sequences encoded by repeat genes suggested that, unlike segment duplication events, the protein sequences encoded by the tandem duplication genes were more similar in *Arabidopsis*, which was supported by the phylogenetic tree. There were 5 of 6 groups of tandem duplications that showed independent branches. However, in soybean, the protein sequences encoded by the segment duplication events were more similar, and 8 of the 22 groups of segment duplications showed independent branches on the phylogenetic tree. No obvious cluster was found in *Lotus* and *Medicago*. These genes with highly consistent sequences are likely to participate in the same biological process in plants.

**Figure 3:**
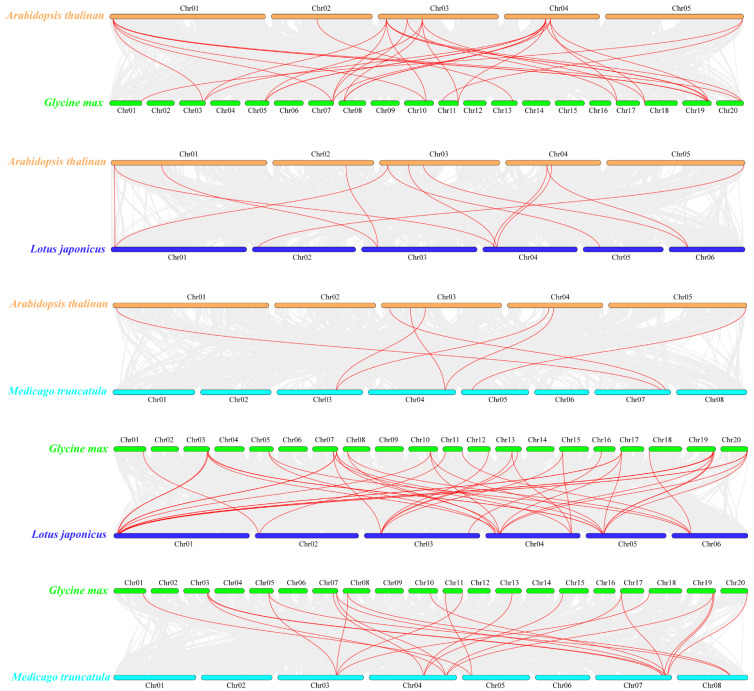
Synteny analysis of *RALF* gene family among four species. Gray lines in the background indicate the collinear blocks within *Arabidopsis* and other plant genomes, while the red lines highlighted the synteny *RALF* pairs.

In order to reveal the evolutionary mechanism of the *RALF* gene family, syntenic relationships were performed between non-legume and legume, as well as determinate and indeterminate nodule plants (Figure 3), respectively. The results showed that a total of 11 non-redundant *AtRALFs* exhibited syntenic relationships with three legume species. Soybean had the most syntenic genes with *Arabidopsis*. There were seventeen *GmRALFs* formed thirty-one syntenic pairs with nine *AtRALFs*, while eight *LjRALFs* formed twelve syntenic pairs with ten *AtRALFs* and five *MtRALFs* formed seven syntenic pairs with seven *AtRALFs* (Table S7). Notably, seven *AtRALFs* (*AtRALF1*, *AtRALF22*, *AtRALF23*, *AtRALF24*, *AtRALF31*, *AtRALF33*, *AtRALF34*) had syntenic pairs throughout all the legume species. These genes may be inherited from their common ancestors. On the other hand, the syntenic relationship between legume plants was significantly stronger than that of non-legume plants and legume plants. Among them, there were 43 syntenic pairs between soybean and *Lotus* consisting of 22 *GmRALFs* and 11 *LjRALFs*. However, the number of syntenic pairs between soybean and *Medicago* was only 23, which was much lower than that between soybean and *Lotus*.

In order to determine the selection pressure of the *RALF* gene family during evolution, the value of non-synonymous to synonymous substitution ratio (Ka/Ks) was used to characterize the evolutionary ability of *RALFs* in duplication events. The results show that all the orthologous *RALF* gene pairs between two species displayed Ka/Ks < 1, indicating that the *RALF* gene family was limited in the evolutionary process.

### 2.5. Expression Profile of GmRALFs in Soybean in Response to Bradyrhizobium Japonium Symbiosis

**Figure 4:**
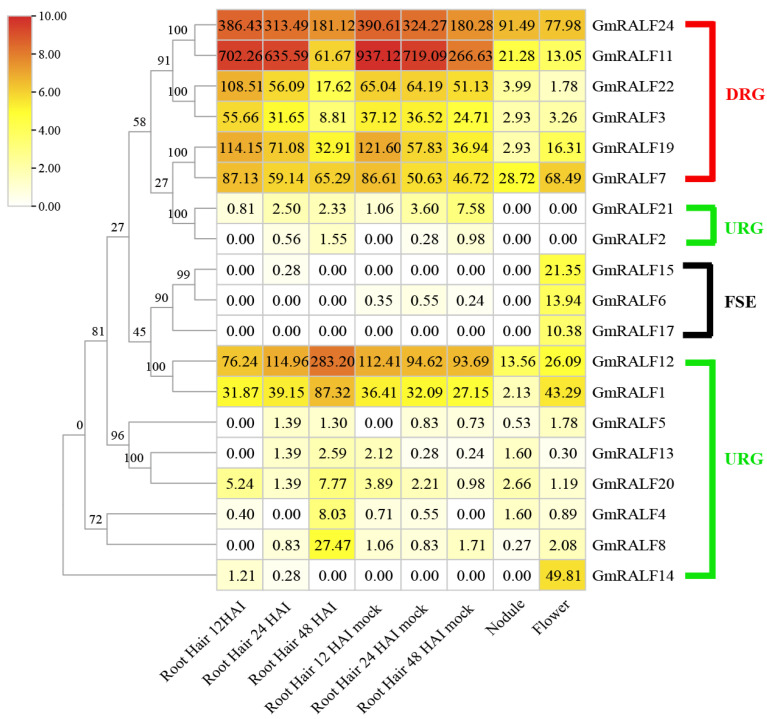
Expression profile of *GmRALFs* in the root hair, nodule and flower. Root hair 12 HAI/Root hair 24 HAI/Root hair 48 HAI and Root hair 12 HAI mock/Root hair 24 HAI mock/ Root hair 48 HAI mock indicated root hairs at 12 h/24 h/48 h after inoculation with or without *Bradyrhizobium japonium* infection, respectively. The real data of Root hair 48 HAI were replaced by Root hair 48 HAI Stripped. The number in the box represented the FPKM value of *GmRALFs*. The colored scale varies from white to red, which indicates low or high levels of gene expression. The unrooted tree was constructed by the neighbor-joining method with 1000 bootstrap replicates.

In order to investigate the mechanism of *RALF* family genes in the early stage of symbiosis, the transcriptome data were obtained from the online transcriptome database *Glycine max* eFP Browser (http://bar.utoronto.ca/efpsoybean/cgi-bin/efpWeb.cgi (accessed on 20 June 2022)), including flowers, root hairs with/without the treatment of *B.japonium* and nodules (Figure 4). 

Apart from five genes (*GmRALF9*, *GmRALF10*, *GmRALF16*, *GmRALF18*, *GmRALF23*) with no corresponding transcriptome data in the database (not shown), three genes (*GmRALF6*, *GmRALF15*, *GmRALF17*) were the only highly expressed ones in flowers. The other *RALFs* (16 of 24) showed varying degrees of expression in underground tissues. Our results showed that all these 19 *RALFs* can be divided into three groups: One was down-regulated genes (DRG), which contains six members, and the expression levels of these genes in root hair were significantly down-regulated by inoculation treatment; another was up-regulated genes (URG), which contained ten members, and the expression levels of these genes in root hair were significantly up-regulated by inoculation treatment, the third one was flower specific expression genes (FSE). These three groups showed an obvious clustering phenomenon in the constructed soybean phylogenetic tree (Figure 4). In addition, the overall expression level of DRG was significantly higher than that of URG in root hair tissues ignoring inoculation and non-inoculation.

From the results of protein sequence similarity comparison, four members (*GmRALF7, GmRALF11, GmRALF19, GmRALF24*) of DRG exhibited higher homology with AtRALF23/33 than that of *GmRLAF2* and *GmRLAF21* in URG, which contribute to plant immunity in *Arabidopsis* [39,52,64]. Additionally, two members (*GmRALF1* and *GmRALF 12*) in URG showed higher sequence similarity with AtRALF34, a protein that regulates pollen tube rupture [28]. Members in FSE showed higher sequence similarity to AtRALF4/19, proteins that are required to maintain pollen tube integrity [27,28]. Furthermore, seven members (*GmRALF1*, *GmRALF2*, *GmRALF4*, *GmRALF8*, *GmRALF12, GmRALF13, GmRALF20*) in URG showed a higher expression level in the root hair tissues at 48 h after inoculation (HAI), compared to 12 HAI and 24 HAI. Previous reports showed that the rhizobia inside the infection thread completed the colonization of cortical cells around 48 h [65,66,67]; therefore, these seven genes in URG may have facilitated the colonization of rhizobia in cortical cells.

Above all, most *RALFs* in soybean are expressed in flower or root hair tissues, and the differential expression pattern in different times suggests that they are not wholly redundant. Whether DRG participates in the process of immunity, URG is involved in the symbiotic process, or FSE specifically regulates flower development, more experimental evidence is needed.

## 3. Discussion

The *RALF* gene family is widely distributed in plants, animals and fungi [30,32,33,34,35]. These secreted peptides act as important signaling peptides in several physiological and developmental processes by strongly binding to its receptor kinases [55,56]. In the past twenty years, studies on the *RALF* gene family in plants mainly focused on non-legume plants, such as *Arabidopsis*, rice, maize, polar and even strawberry [51,54]. The symbiotic nitrogen (N)-fixation system formed by legume and rhizobia fixed up to 200 million tonnes of N per annum, plays a crucial role in N fixation and cycling [16]. The *RALFs* have been identified in the growth and development of pollen tubes and root hairs [56]. Interestingly, the growth of the infection thread during the early stages of symbiosis between legume and rhizobia is a polar growth mode similar to pollen tubes and root hairs [68]. However, it is not clear whether *RALFs* were involved in the invasion of rhizobia bacteria (the polarized growth of infection thread during symbiotic). This study presented a comprehensive analysis of the *RALF* gene family in one non-legume species (*Arabidopsis*) and three legume species (soybean, *Lotus* and *Medicago*). The results of our study provide detailed information about the *RALF* gene family in legume–rhizobia symbiosis, especially in soybean-*rhizobium*.

This study identified 41, 24, 17 and 12 *RALFs* in *Arabidopsis*, soybean, *Lotus* and *Medicago* genomes, respectively (Table S1). The gene number of *RALFs* varied significantly between species and is not consistent with the previous study [46,51,52,53,69]. A negative correlation between the genome size, total gene number and the gene number of *RALFs* were detected in our results, which was also observed in a previous study [52]. For example, soybean has the biggest genome size (1115 Mb) and the largest gene number (56,044) of the four species in this study, but the number of *RALFs* in soybean is significantly less than *Arabidopsis* (~135 Mb; 27,655), which has the smallest genome size and the fewest number of genes. Even though a previous study reported that the divergence time of Fabales was earlier than Brassicales [61], whether there is a certain connection between gene expansion and species evolution needs more evidence. Typical basic protein possessed a negative (basic) charge and in the basic range with an average pI of 8.37 [70,71]. In this study, the average pI values of RALF precursor proteins in legume species are lower than that in non-legume species, indicating that the alkaline activity of RALF proteins in legume species is weaker.

Phylogenetic analysis revealed that 94 *RALFs* were divided into two groups: Group A and Group B, which included three (Group A1–A3) or seven (Group B1–B7) subgroups, respectively, with a number of genes ranging from three (Group A1) to twenty-two (Group A3). Among these subgroups, Group A2 and Group B7 were composed of single species, respectively. It is speculated that these *RALFs* from a single species may be related to species-specific growth regulation. Genes in the same cluster may play analogous biological functions despite their origins [72]. Notably, each subgroup had specific conservation in functional domains, which will support the reliability of our classification results (Table S5). For example, the *RALFs* in the whole of Group A did not contain the RR motif, suggesting that the proper cursor encoded by *RALFs* in this cluster cannot be recognized and cleaved by S1P.

The acquisition of alkalinization activity and immune activity of RALF protein depended on the processing of precursor peptides by a plant subtilisin-like serine protease, S1P, and the conservation of multiple sites on the mature RALF peptide sequences ensures its alkalinization activity [38,47,50]. By comparing the distribution differences of RR motif, -YIXY- motif, Leu-11 and the residue of four conserved cysteines in RALF proteins of non-legume species and legume species, those important motifs/residues related to alkalinization activity were more conservative in legume species, especially in soybean (Table S6). For example, the RR motif existed in 83.33% of soybean RALF precursor proteins, which has been proven to be essential for proper maturation and release of RALF active peptides. While only 52.94% and 66.67% RALFs contained this motif in *Medicago* and *Lotus*. The lowest proportion occurred in *Arabidopsis*; only 29.27% *RALFs* have experimental evidence in *Arabidopsis*, all containing RR motifs. For example, *AtRALF4*, *AtRALF19*, *AtRALF34*, *AtRALF1* and *AtRALF23*, which have been reported in the regulation of pollen tubes, hypocotyls and roots [28,50,73]. Another example is *AtRALFL8*, which lacks the RR motif. Although 35S::AtRALFL8 lines produced a higher expression than the wild-type and had a severely stunted phenotype, non-transfer DNA insertion lines were available for AtRALFL8 [74]. Stegmann et al. [38] reported that the mature peptides cleaved by S1P can facilitate the ligand-induced complex formation of the immune receptor kinases FER and flagellin sensing 2 (FLS2) with their co-receptor brassinosteroid insensitivity 1-associated kinase 1 (BAK1) to inhibit plant immunity. There were 20 RALFs in soybean that can be cleaved by S1P to form active peptides, but which genes were involved in immune regulation during the symbiosis process still need to be further confirmed. Considering that the RR motif is essential for proper maturation and release of RALF active peptides, it is reasonable to take the proportion of conservation functional domains on mature peptide sequences only when the RR motif is present into account. However, previous studies have not paid attention to this [39,48].

The expansion of the gene family depends on gene replication. Tandem duplication, segmental duplication and transposition are the three main modes of gene replication [62]. In plants, tandem duplication events are considered as the main way for the expansion of the *RALF* gene family [52,53,54,69]. However, this study showed that tandem duplication was the only expansion mode in *Arabidopsis*, segmental duplication played a dominant role in the expansion of the legume *RALFs* (Figure 2). In *Arabidopsis*, there were six pairs of tandem duplications and five pairs of segmental duplications in the *RALF* gene family. However, in soybean, *Medicago* and *Lotus*, the frequency of segmental duplication events was higher than tandem duplication. In particular, in the soybean genome, twenty-two pairs of segmental duplications and only one pair of tandem duplication were identified. We speculate that in the soybean genome, most of the *RALFs* come from a common ancestor gene. Furthermore, some *RALFs* in soybean have undergone segmental duplication more than once. This phenomenon may be related to the two genome duplication events in soybean [75]. Meanwhile, in the synteny analysis, soybean has the most collinear gene pairs with other species. This is probably beneficial for the large genome size and numerous gene numbers in soybean. It is worth noting that although the number of genes in *Lotus* genome is about half of that in *Medicago*, more collinear gene pairs were detected in the former when synteny analysis was carried out with soybean. Considering that legumes produce two different types of nodules in symbiosis with rhizobia: determinate type of nodules (soybean and *Lotus*) and indeterminate type of nodules (*Medicago*), we suspect that *RALFs* may have more similar functions in species with the same nodule type. Our analysis provides evidence for a comprehensive understanding of the evolution and expansion of the *RALF* gene family in different species.

As an important economic and food crop, the symbiotic nitrogen fixation between soybean and rhizobia is crucial for yield improvement, and the successful establishment of the symbiotic relationship is a prerequisite for this. Previous studies have shown that inoculation treatment could induce the expression of early marker genes related to nodulation in soybean, such as *GmNINa/b*, *GmNSP1a/1b*, *GmNSP2a/2b*, *GmNFR5a/b* and *ENOD40*. These genes were up-regulated within 72 h after inoculation [76,77,78,79]. On the other hand, immune-related genes, such as the PR family genes, were significantly suppressed in expression upon inoculation. In soybean species, the expression levels of 16 *GmRALFs* were changed after inoculation. They were six DRG and ten URG identified in the root hair tissues after inoculation. Meanwhile, seven members (*GmRALF1*, *GmRALF2*, *GmRALF4*, *GmRALF8*, *GmRALF12*, *GmRALF13*, *GmRALF20*) in URG showed a higher expression level in the root hair tissues at 48 h after inoculation, compared to 12 HAI and 24 HAI. Previous reports have shown that the rhizobia inside the infection thread completed the colonization of cortical cells around 48 h [65,66,67]. Whether these 16 *GmRALFs* were involved in symbiosis, and whether these 7 *GmRALFs*, which were significantly upregulated at 48 h were related to the release of rhizobia in the cortical cells, still need further experimental verification.

In summary, this study completed a comparative analysis of the *RALF* gene family in non-legume and legume plants. The comparison results showed that RALF peptides in legume plants were more conserved in main functional domains. The analysis of phylogenetic trees showed that there was a differentiation between RALFs from different species. Further analysis of the expansion mode of the gene family shows that the *RALF* gene family in legumes adopts an inconsistent expansion mode in non-legume. This is also the first report to reveal the role of the *RALF* gene family in symbiotic nitrogen fixation. Our research will contribute to a deeper understanding of the biological function of this family gene in the early stage of symbiotic nitrogen fixation.

## 4. Materials and Methods

### 4.1. Identification of RALF Family Members in Legume and Non-Legume Genomes

In order to search *RALFs* in legume plants more comprehensively, the number of *AtRALFs* was confirmed first, which were used as seed genes in our subsequent analysis. A keyword search approach was used to exploit new *AtRALFs* in the *Arabidopsis* Information Resource (TAIR) database (https://www.arabidopsis.org (accessed on 20 June 2022)) [80], NCBI (https://www.ncbi.nlm.nih.gov (accessed on 20 June 2022)), Pfam (https://pfam.xfam.org (accessed on 20 June 2022)) database [81] and RALF domain was verified in all putative proteins by using SMART (http://smart.embl-heidelberg.de (accessed on 20 June 2022)) [82] and CDD (https://www.ncbi.nlm.nih.gov/cdd (accessed on 20 June 2022)) databases [83]. 

Next, all the legume genome and annotation files were downloaded from Ensembl (https://asia.ensembl.org/index.html (accessed on 20 June 2022)) and Phytozome v13 (http://www.phytozome.net (accessed on 20 June 2022)) database [84]. Forty-one AtRALF protein sequences as the query sequences were used to extract the most representative legume RALF protein sequences in soybean, *Lotus* and *Medicago* by the TBtools software (https://github.com/CJ-Chen/TBtools/releases (accessed on 21 June 2022)) [85]. Pfam [81], SMART [82] and CDD websites [83] were used to confirm the RALF domains in all the candidate legume RALF proteins.

Considering that the *Wm82.a2.v1* version is still commonly used as the reference genome by most studies, we ultimately chose the *Wm82.a2.v1* version as our reference genome. At the same time, we noticed that in Liu’s article, Glyma.08G248700 (GmRALF12) was mistakenly included in the *RALF* gene family, because this protein did not have the RALF domain, which is one of the most fundamental criteria for identifying the *RALF* gene family. Therefore, we renamed all genes in this study.

The DNA and protein molecular characterization of *RLAFs*, including DNA Sequence length, protein length, protein molecular weight, pI, subcellular localization and signal peptide prediction, were obtained from TAIR [80], SoyBase (https://soybase.org (accessed on 21 June 2022)) [86], Phytozome v13 [84], ExPASy (https://web.expasy.org/protparam (accessed on 21 June 2022)), UniProt (https://www.uniprot.org (accessed on 21 June 2022)) [87], WoLF PSORT (https://www.genscript.com/wolf-psort.html?src=leftbar (accessed on 21 June 2022)) [88], cNLS Mapper (http://nls-mapper.iab.keio.ac.jp/cgi-bin/NLS_Mapper_form.cgi (accessed on 21 June 2022)) and iPSORT (https://ipsort.hgc.jp (accessed on 21 June 2022)) [89].

### 4.2. Sequence Alignments and Phylogenetic Tree Construction of RALF Members

To analyze the conservation of the protein sequence, 94 RALF proteins with full-length sequences were used for multiple sequence alignment. DNAMAN version 8 was used in this analysis. The evolutionary relationship of the RLAF family in non-legume and legume species was analyzed by using neighbor-joining (NJ) methods in MEGA 6 (https://www.megasoftware.net/ (accessed on 21 June 2022)) with 1000 bootstrap replicates [90]. The phylogenetic tree was beautified and visualized by the iTOL website (https://itol.embl.de/ (accessed on 21 June 2022)) [91].

### 4.3. Chromosome Locations and Synteny Analysis

Due to the different number of genes and genome sizes among different species, it is important to set an appropriate genetic interval to better demonstrate the distribution characteristics of genes on chromosomes. The size of the genetic interval was adequately considered based on the number of genes within the interval, and the interval with too many (gene number > 100, hereditary interval > 500 kb) or too few (gene number < 10, hereditary interval < 100 kb) genes is not considered appropriate. Therefore, different species with different genetic intervals in this study. The genetic intervals for *Arabidopsis*, soybean, *Lotus* and *Medicago* were set as 100 kb, 500 kb, 500 kb and 300 kb, respectively. Next, we obtained the species-specific paralogous gene pairs through genome comparison and visualized the chromosomal locations of the *RALFs*. 

In order to confirm the evolutionary relationship between the *RALF* gene family in legume and non-legume species, a synteny analysis and selection pressure were conducted on *RALFs* of different species. A genome alignment between soybean, *Lotus*, *Medicago* and *Arabidopsis* to identify the expansion of the *RALF* gene family between legume and non-legume species. The genome alignment between *Lotus*, *Medicago* and soybean helped to identify the evolutionary pattern of *RALF* genes in legume species. All the work was completed using TBtools software [85].

### 4.4. Expression Analysis of Soybean RALF Genes

To better understand the function of *RALFs* during symbiotic nitrogen fixation, all the transcriptome data of *RALFs* in root hairs, nodule and flowers were downloaded from online transcriptome database *Glycine max* eFP Browser [78,92]. TBtools software [85] was used to draw a heat map according to the expression profile of all the soybean *RALFs*. Considering the accuracy of experimental data, the 48 HAI transcriptome data was replaced with 48 HAI Stripped transcriptome data. The phylogenetic tree of soybean RALFs was constructed by using MEGA version 6 [90].

## 5. Conclusions

In this study, we performed the first comparative analysis of *RALFs* in non-legume species and legume species and described a detailed analysis of their molecular features, phylogenetic relationship, motifs/resides composition and conservation and chromosome distribution. We also characterized the expression patterns of *GmRALF* genes in response to rhizobia. The differential expression of *GmRALFs* in root hair tissues has a tendency to regulate symbiosis. Our findings provide novel insights into the understanding of the role of *RALFs* in legume and nodule symbiosis.

## Figures and Tables

**Table 1:** The Statistics of RALF-Conserved Motif/Residues Under the Presence of RR Motif.

Species	*Arabidopsis*	Soybean	*Lotus*	*Medicago*
Gene number	41	24	17	12
Predicted S1P cleavage site (%)	29.27	83.33	52.94	66.67
YI/LXY motif (%)	29.27	70.83	47.06	58.33
Leu-11	26.83	83.33	52.94	66.67
Conserved Cysteine 1 (%)	26.83	79.17	52.94	66.67
Conserved Cysteine 2 (%)	26.83	66.67	41.18	50.00
Conserved Cysteine 3 (%)	29.27	83.33	52.94	66.67
Conserved Cysteine 4 (%)	29.27	79.17	52.94	66.67
Mean Percentage (%)	28.05	77.08	50.00	62.50

## Data Availability

The datasets used and/or analyzed during the current study are available from the corresponding author on reasonable request. However, most of the data are shown in Supplementary Files.

## Supplementary Materials

The following supporting information can be downloaded at: https://www.mdpi.com/article/10.3390/ijms24108842/s1. References [58,59,60,73,80,93] are cited in the supplementary materials.

## Abbreviations

RALF	Rapid alkanization factor
At	*Arabidopsis thaliana*
Gm	*Glycine max*
Lj	*Lotus japonicus*
Mt	*Medicago truncatula*
MW	Molecular weight
Pi	Isoelectric point
Aa	Amino acid
CDS	Coding sequences
Ka/Ks	Non-synonymous/synonymous substitution
DRG	Down-regulated genes
URG	Up-regulated genes
FSE	Flower specific expression genes
Mb	Megabases
Kb	Kilobase
